# Evaluation of the effect of a gel made with amniotic fluid formulation on the healing of diabetic foot ulcers: A triple-blind clinical trial

**DOI:** 10.3389/fpubh.2022.1025391

**Published:** 2022-12-15

**Authors:** Fatemeh Niami, Shahram Molavynejad, Ali Asghar Hemmati, Darioush Bijan Nejad, Leila Yazdanpanah, Nader Shakiba Maram, Amal Saki Malehi, Mandana Mahmoudi

**Affiliations:** ^1^Nursing Care Research Center in Chronic Diseases, School of Nursing and Midwifery, Ahvaz Jundishapur University of Medical Sciences, Ahvaz, Iran; ^2^Marine Pharmaceutical Science Research Center, School of Pharmacy, Ahvaz Jundishapur University of Medical Sciences, Ahvaz, Iran; ^3^Cellular and Molecular Research Center, Medical Basic Sciences Research Institute, Ahvaz Jundishapur University of Medical Sciences, Ahvaz, Iran; ^4^Diabetes Research Center, Health Research Institute, Ahvaz Jundishapur University of Medical Sciences, Ahvaz, Iran; ^5^Nanotechnology Research Center, Ahvaz Jundishapur University of Medical Sciences, Ahvaz, Iran; ^6^Pain Research Center, Ahvaz Jundishapur University of Medical Sciences, Ahvaz, Iran; ^7^Obstetrics and Gynecologic Department, Mehr Hospital, Ahvaz, Iran

**Keywords:** diabetic foot ulcer, wound healing, amniotic fluid, nursing care, Iran

## Abstract

**Aim:**

The aim of this study was to evaluate the effect of a gel made with amniotic fluid (AF) formulation on wound healing in diabetic foot ulcers.

**Methods:**

This clinical trial was performed on 92 type 2 diabetic patients referring to the Diabetes Clinic of Golestan Hospital of Ahvaz, southwest of Iran in 2019–2020. Patients were randomly divided into three groups of intervention and one placebo group. The wounds of the three intervention groups were dressed with gauze impregnated with an AF formulation gel while wounds of the control group were dressed with plain gauze without any topical agent. Chi-square tests and generalized estimating equations (GEE) with a significance level of 0.05 were used to analyze the data.

**Results:**

At the end of the eighth week of intervention, there was a statistically significant difference among the four groups in terms of wound grade, wound color, condition of the tissues surrounding the wound, the overall condition of the wound, and the duration of wound healing (*P* < 0.05).

**Conclusions:**

Based on our experience with the patients in the present study, we believe that AF represents a useful and safe option for the treatment of chronic diabetic foot ulcers.

**Clinical trial registration:**

https://en.irct.ir/trial/51551, Identifier: IRCT20201010048985N1.

## Introduction

Diabetes is one of the health problems in the world today and its prevalence in adults is currently 6.4% which is estimated to reach 17.7% in 2030 (1). Common diabetes comorbidities including peripheral neuropathy, ischemia, callus formation, deformity, edema and peripheral arterial disease (PAD) are among the well-established risk factors for diabetic foot ulcers (2). Diabetic foot ulcers are the most common cause of hospitalization of diabetic patients (1, 3). Diabetic foot ulcers are more common in men than women, and type 2 diabetic foot ulcers are more likely to develop than those caused by type 1 diabetes. According to the literature, the global prevalence of diabetic foot ulcers is 6.6%. It has also been reported that the prevalence of diabetic foot ulcers in Asia is 5.5% (4). In Iran, this prevalence has been reported to be between 4–10%, and the rate of lower limb amputation in diabetic patients is 15 times higher than that in otherwise normal people (5).

In addition to long-term hospitalization, foot ulcers and amputations increase treatment costs, reduce quality of life and self-esteem increase patient dependence, and can even lead to patient mortality (6). Therefore, prevention of foot ulcers and amputations and identification of diagnostic methods and timely and effective treatment can increase the quality of life of these patients and reduce their treatment costs (7). Common treatments for diabetic foot ulcers include proper glycemic control, appropriate antibiotic therapy, debridement of necrotic tissue, pressure-relieving strategies restoring pulsatile blood flow, negative pressure wound therapy (8), high-pressure oxygen (9), and wound closure with a vacuum generator (10).

Regenerative medicine, a commonly used phrase in the field of chronic wound management, is the “process of replacing or regenerating human cells, tissues, or organs to restore or establish normal function” (11). Recent studies show that amniotic fluid (AF) contains a considerable quantity of multipotent mesenchymal, hematopoietic, neural, epithelial, and endothelial stem cells (12). AF also contains factors that are involved in wound healing. These include prostaglandins, carbohydrates, peptides, lipids, lactate, amino acids (e.g., glutamine and arginine), proteins (e.g., lactoferrin), enzymes, minerals (e.g., iron and zinc) and hormones (e.g., growth hormone and prolactin). In human amniotic fluid, transforming growth factor (TGF)-α, TGF-β1, platelet-derived growth factor (PDGF) and fibroblast growth factor (FGF) seem to stimulate the cutaneous fibroblast proliferation (13, 14). Many of the substances that constitute the innate immune system have been identified in AF and have been shown to have significant antimicrobial properties, including α-defensins (human neutrophil defensins 1–3), calprotectin, secretory leukocyte protease inhibitor, psoriasin (S100A7), lactoferrin, lysozyme, bactericidal/ permeability-increasing protein, and cathelicidin (15). These potent antimicrobials have been shown to have broad-spectrum activity against bacteria, fungi, protozoa, and viruses (14). *In vitro* study showed that AF enhanced collagenase activity but inhibited activity of cathepsin, elastase, and hyaluronic acid (16). Alamouti et al., found that amniotic membrane extract heals diabetic foot ulcers (17). Also, a study by Abdo, in 2016 showed that the use of dehydrated amniotic membrane allograft is effective in healing diabetic foot ulcers (18).

AF is a biological substance that is disposable after childbirth and can be obtained for free after making arrangements with the related healthcare facilities. However, to the best of our knowledge, no study has yet examined the effect of using AF on healing of diabetic foot ulcers. Therefore, the present study was conducted to investigate the effect of AF on wound healing in diabetic foot ulcers.

## Materials and methods

### Design

This triple blind clinical trial was conducted in 2019–2020.

### Study population

The study population included all patients with grades 1 and 2 diabetic foot ulcers referred to Diabetes Clinic of Golestan Hospital of Ahvaz and physician offices in Ahvaz, Iran. Considering recovery ratios of 73.33% and 13.33% and assuming a significance level of 0.05 a power of 90%, and an attrition rate of 10%, the final sample size was 92. The patients were assigned into four groups (A, B, C, D) using the randomized permuted block design, which included three intervention groups and one control group (23 patients in each group). The intervention of this study took 8 weeks. The participants were eligible to participate in the study if they: had grade 1 or 2 diabetic foot ulcer according to Wagner system classification, were 18 years old and older, did not smoke or use drugs, did not take medications such as corticosteroids or immunosuppressive and toxic agents that may interfere with wound healing, lack of concomitant diseases such as cancer, vasculitis, renal and hepatic failure, did not have advanced heart failure that may interfere with wound healing process, and were able to fill out the informed consent form. Patients were excluded from the study if they: had active wound infection requiring intravenous antibiotics or gangrenous ulcer requiring amputation, evidence of ischemic, venous, or traumatic lesions, malignancy in the wound area or any malignancy in the patient. Patients who either did not refer to the center for follow up, changed the dressing more than two consecutive times, participated in another research project, or had any sensitivity to AF were also excluded.

For blinding in this study, the gel with AF formulation in three percentages of 5, 10 and 15, and the placebo were produced by a pharmacologist. The dosage was determined using labels A, B, C, D, of which only the pharmacologist was aware. The gel with AF formulation was administered to the patients by the lead researcher, and the wound healing evaluation was performed by a physician who was blind to group assignment in the diabetes clinic.

### Instruments

Data collection tools in this study included a form including the patients' demographic and clinical characteristics, Wagner wound classification system and a checklist for evaluating the healing of diabetic foot ulcer. The demographic form included age, gender, occupation, marital status, educational attainment, comorbidities, type of diabetes treatment, ulcer location and body mass index.

The Wagner system assesses ulcer depth and the presence of osteomyelitis or gangrene using the following grades: grade 0 (pre-or post-ulcerative lesion), grade 1 (partial/full thickness ulcer), grade 2 (probing to tendon or capsule), grade 3 (deep with osteitis), grade 4 (partial foot gangrene), and grade 5 (whole foot gangrene) (19).

Ulcer healing assessment scale evaluates on a weekly basis 4 ulcer parameters including color, surrounding tissues, drainage, and degree, as well as overall ulcer status. Based on this scale, the maximum score for each parameter is 100, and the overall ulcer status scores range from 50 to 400. Based on this scale, higher scores represent better healing (Table 1) (20).

**Table 1:** Diabetic foot ulcer healing assessment scale.

**Total score**	**Ulcer parameters**	**Distribution of scores**																
**100**	**Degree**	**Stage**	**0**	**1**	**2**	**3**	**4**	**5**	**6**	**7**	**8**	**9**	**10**	**11**	**12**	**13**	**14**	**15**
		**Score**	**100**	**90**	**90**	**80**	**75**	**65**	**65**	**55**	**50**	**40**	**40**	**30**	**25**	**15**	**15**	**10**
100	Color	Center	Total healing			Red			Yellow			Necrotic			Necrotic + Red			
			50			40			30			20			10			
		Surroundings	Total healing			Red			Yellow			Necrotic			Necrotic + Red			
			50			40			30			20			10			
100	Surrounding tissues	Color	Normal			Red			Pale			Cyanotic			–			
			25			20			15			10			–			
		Hotness	Yes			No			–			–			–			
			0			25			–			–			–			
		Edema	Yes			No			–			–			–			
			0			25			–			–			–			
		Sense	No			Decreased			Yes			–			–			
			0			15			25			–			–			
100	Drainages	Color	Without drainages			Serosal			Bloody			Yellow			Green			
			40			30			30			20			10			
		Odor	No			Yes			–			–			–			
			20			0			–			–			–			
		Amount	Without drainages			Low			Moderate			Much			–			
			40			20			20			10			–			
Total score (total healing) = 400 (more score= more healing)																		

At the end of the eighth week, the last score (score of week 8) was compared to the first score (score of week 0) and status of the ulcer was defined as full recovery, partial recovery, no recovery, and deterioration.

Full recovery: In case that total scores of the ulcer were equal to 400 according to the checklist.

Partial recovery: In case that total scores of the ulcer were increased at least 30-fold compared to the initial score.

No recovery: In case the score did not change compared to the initial score or its increase was below 30-fold.

Worsening: In case the score decreased 10-fold compared to the initial score (20).

#### Intervention

In this study, access to AF was made possible after arrangements were made with Ahvaz Neurogenic Laboratory. In the neurogenic laboratory, pregnant women who were in their 14th to 20th weeks of pregnancy, after being diagnosed by a gynecologist for amniocentesis, underwent sterile amniocentesis under ultrasound guidance, and about 20–30 ml fluids were drawn in each amniocentesis. It should be noted that the AF provided to the research team was obtained from pregnant mothers whose viral test was negative for markers including HIV, HBs Ag, and HCVab. The obtained fluid was taken to Jundishapur University Diabetes Center using a cold box, and it was stored in the center's refrigerator at 1 °C. The AF was centrifuged at 1100 g for 8 min. The supernatant fluid was used to prepare the gel formulation.

2-3-1 AF gel formulation.

I order to find the most effective dose of the drug according to advice of the pharmacologist of the research team, the AF gel was prepared using 5, 10, and 15 % w/w gel base. The gel base consisted of 15% propylene glycol, 2% hydroxy propyl methyl cellulose (HPMC), and distilled water which are non-active ingredients of the formulation and have no inflammatory or immunological reactions. Therefore, the 5, 10 and 15% gels were prepared as w/w method. That is, the 5% AF gel consisted of 5 g of AF and 95 g of gel base, the 10% AF gel consisted of 10 g of AF and 90 g of gel base, and the 15% AF gel consisted of 15 g of AF and 85 g of gel base. The placebo consisted only of gel base. In sum, in the intervention groups, the gel contained AF in 5, 10 and 15 percentages, while the gel (placebo) used in the control group contained all the gel ingredients except AF.

To perform the intervention, the patients' wounds were first examined by a physician, and if necessary, wound debridement was performed. Then, in each group, the wound and the surrounding tissue were washed with 0.9% normal saline and then dried with sterile gauze. As a routine procedure for new topical drugs, to control the possible allergic effects of preparation, a skin patch test was performed on each patient as follows. We put some of the prepared formula on disk-like plates called patches and stuck the patch on the inside of the forearm or the arm. After a certain period of time, we removed the patch. Any evidence of contact dermatitis (itching, swelling, redness, induration, etc.) led to the patient's exclusion from the study population.

**Figure 1:**
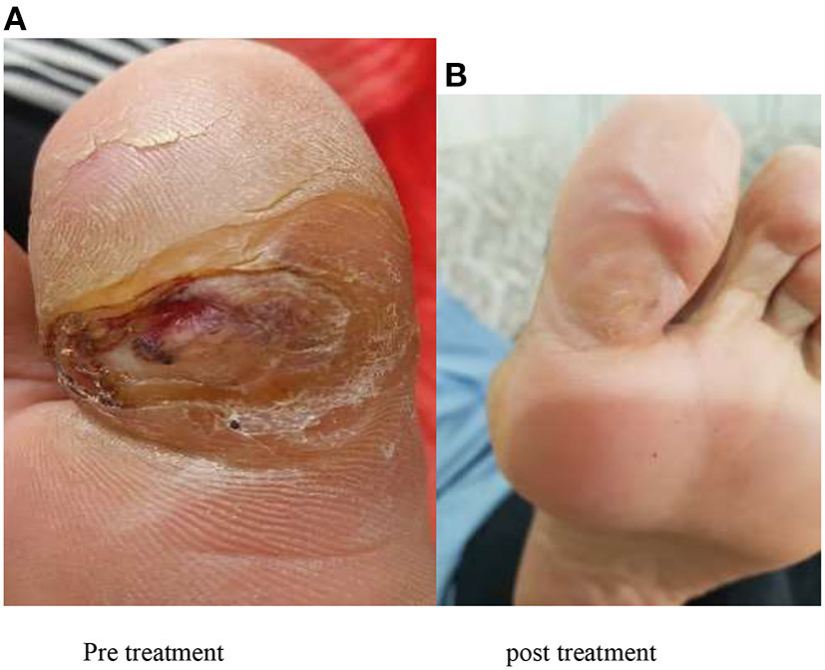
Picture shows an example of wound healing. **(A)** Pre-treatment. **(B)** Post-treatment.

After washing the wound, the gel containing AF formulation was placed on the wound so that the whole wound surface was impregnated with the amniotic gel and then a sterile dressing was applied. Patients or their companions were given the amniotic gel and were taught how to use it and perform the correct dressing. It was recommended that the dressing be changed every 24 hours, and the patients were advised to refer to the diabetes clinic once a week for evaluation of wound healing (Figure 1 shows an example of wound healing).

### Ethical consideration

This clinical trial was registered in the Iranian Registry of Clinical Trials (Ref. ID: IRCT20201010048985N1) and approved by the Ethics Committee of Ahvaz Jundishapur University of Medical Sciences (Ref. ID: IR.AJUMS.REC.1399.516). Participants were assured that they would be given any information they need in case of any ambiguities, that they would not be charged for the treatment, and that their information would be used only for research purposes and would be kept confidential.

### Data analysis

After collecting and scoring the data, data analysis was done based on GEE analysis and chi-square test using SPSS version 22. The significance level of the tests was set at *P* < 0.05.

## Results

The final analysis was performed on data obtained from 83 patients, of whom 44 (53%) were female and 39 (47%) were male. Most patients (51.2%) were in the age group of 40–49 years. With regard to occupation, 48.2% were housewives, 24.1% were office workers, 8.4% were self-employed, and 19.3% were retired. Also 83.1% of the patients were married, 8.4% were widow, 7.2% were single, and 1.3% were divorced. As far as education was concerned, 26.5% of the patients were illiterate, 37.3% did not have a high school diploma, 31.4% had a high school diploma, and 4.8% had a university degree. In terms of the ethnicity of the participants, the highest frequency (55.4%) of patients were Arabs while the lowest frequency 1.2% (n = 1) were related to other ethnicities. With respect to body mass index, 51.3% of the patients had a body mass index of 25–29. According to Table 2, 29.1% of the patients' wounds in the 4 groups were on the toes while wounds on the whole foot (1.2%) were the least frequent ones. Also, most patients (51.8%) were diagnosed with grade 2 ulcers according to Wagner classification. Based on chi-square test, there was no significant difference between the 4 groups in terms of demographic information (*P* > 0.05) (Table 2).

**Table 2:** Frequency distribution and percentage of demographic information of the four groups of patients participating in the study.

**Variable**	**Classification**	**Group 10%, *N* (%)**	**Group Placebo, *N* (%)**	**Group 5%, *N* (%)**	**Group 15%, *N* (%)**	*P*-value*
Sex	Female	12 (57.1)	12 (57.1)	9 (42.9)	11 (55)	0.756
	Male	9 (42.9)	9 (42.9)	12 (57.1)	9 (45)	
Age (year)	18–39	3 (14.3)	1 (4.7)	0 (0)	1 (5)	0.17
	40–49	9 (42.9)	14 (66.8)	8 (38.2)	11 (5)	
	50–59	5 (23.8)	5 (23.8)	11 (52.4)	8 (40)	
	60–69	2 (9.5)	0 (0)	1 (4.7)	0 (0)	
	70>	2 (9.5)	1 (4.7)	1 (4.7)	0 (0)	
Occupation	Housewife	8 (38.1)	12 (57.1)	9 (42.9)	11 (55)	0.11
	Employed	8 (38.1)	3 (14.3)	8 (38.1)	1 (5)	
	Retired	2 (9.5)	4 (19.1)	3 (14.3)	7 (35)	
	Unemployed	3 (14.3)	2 (9.5)	1 (4.7)	1 (5)	
Marital status	Single	2 (9.5)	2 (9.5)	1 (4.8)	1 (5)	0.49
	Married	18 (85.7)	17 (81)	16 (76.2)	18 (90)	
	Divorced	0 (0)	0 (0)	0 (0)	1 (5)	
	Widow	1 (4.8)	2 (9.5)	4 (19)	0 (0)	
Education	Illiterate	4 (19)	4 (19)	9 (42.9)	5 (25)	0.27
	Primary school	6 (28.6)	12 (57.2)	5 (23.8)	8 (40)	
	High school diploma	10 (47.6)	5 (23.8)	5 (23.8)	6 (30)	
	University degree	1 (4.8)	0	2 (9.5)	1 (5)	
Body Mass Index	18–24	6 (28.6)	10 (47.6)	10 (47.6)	9 (45)	0.86
	25–29	13 (61.9)	10 (47.6)	9 (42.9)	10 (50)	
	30–35	2 (9.5)	1 (4.8)	2 (9.5)	1 (5)	
Ulcer location	Sole	5 (23.8)	5 (23.8)	7 (33.3)	5 (25)	0.63
	Heel	3 (14.3)	5 (23.8)	7 (33.3)	5 (25)	
	Toe	7 (33.3)	8 (38.2)	2 (9.5)	8 (40)	
	Foot dorsum	6 (28.6)	3 (14.2)	5 (23.8)	2 (10)	
Wound grade	Grade 1	11 (52.4)	10 (47.6)	11 (52.4)	8 (40)	0.77
	Grade 2	10 (47.6)	11 (52.4)	10 (47.6)	12 (60)	

^*^Chi-square test.

According to the results of GEE analysis, the effect of time was significant (*P*-value < 0.001), with the mean scores in the placebo, Group 5% and Group 10% having an upward trend. However, this trend first increased in the Group 15% and had the highest mean in the fourth week but then decreased. Also, between-group comparison showed that Group 5% was significantly different from the control group (*P*-value < 0.001), but the Groups 10 and 15% were not.

Table 3 shows the mean of wound color scores of patients in the 4 groups over a period of 0–8 weeks. According to the results of GEE analysis, the effect of time was significant (*P*-value < 0.001), with the mean scores in the four groups having an almost upward trend, but this trend in the control group and the Group 5% was initially upward until it reached its peak at the seventh week and then it became downward in Week 8. Also, between-group comparison shows that the treatment Group 5% was significantly different from the control group (*P*-value = 0.004), but the Groups 10 and 15% were not.

**Table 3:** Comparison of mean and standard deviation of ulcer parameters and total ulcer status from week 0 to 8 in 4 group.

		**Group 10%**	**Group 5%**	**Group 15%**	**Placebo**	
**Ulcer parameters**	**Time**	**Mean ±SD**	**Mean ±SD**	**Mean ±SD**	**Mean ±SD**	P*(effect of time)
Wound degree	Week 0	72.27 ± 8.96	74.31 ± 6.41	72.31 ± 4.41	71.26 ± 7.42	<0.001
	Week 1	74.77 ± 6.62	74.77 ± 6.98	73.40 ± 6.24	72.04 ± 6.66	
	Week 2	79.09 ± 7.70	80.68 ± 7.44	74.54 ± 6.52	75.45 ± 6.52	
	Week 3	83.86 ± 7.38	81.66 ± 6.95	76.56 ± 7.92	80.22 ± 6.63	
	Week 4	85.95 ± 7.51	84.00 ± 7.53	87.00 ± 8.80	83.18 ± 5.46	
	Week 5	88.94 ± 6.14	88.15 ± 7.11	77.85 ± 7.83	87.95 ± 5.49	
	Week 6	90.88 ± 4.04	89.06 ± 6.11	77.85 ± 7.51	91.50 ± 4.61	
	Week 7	94.68 ± 2.86	92.33 ± 6.77	79.04 ± 7.68	94.11 ± 6.64	
	Week 8	97.14 ± 3.23	93.18 ± 7.50	79.52 ± 7.89	95.31 ± 8.84	
*P*-value** (effect of treatment)		0.19	<0.001	0.54	–	
Wound color	Week 0	66.36 ± 14.65	70.68 ± 7.91	67.95 ± 11.30	77.67 ± 14.64	**<0.001**
	Week 1	69.09 ± 13.76	71.36 ± 8.47	70.00 ± 6.86	66.81 ± 13.67	
	Week 2	73.86 ± 13.96	77.27 ± 8.12	71.13 ± 9.24	70.45 ± 11.43	
	Week 3	79.77 ± 10.96	79.52 ± 7.56	72.95 ± 9.46	75.68 ± 10.94	
	Week 4	82.61 ± 11.13	83.80 ± 9.86	74.54 ± 9.37	81.13 ± 8.15	
	Week 5	85.87 ± 8.70	87.93 ± 8.39	74.52 ± 8.35	83.18 ± 17.49	
	Week 6	89.11 ± 5.22	88.12 ± 7.93	75.00 ± 7.58	90.25 ± 5.49	
	Week 7	92.80 ± 5.46	91.00 ± 8.25	79.66 ± 7.79	92.94 ± 4.69	
	Week 8	96.42 ± 4.12	90.90 ± 8.89	79.90 ± 7.98	79.18 ± 3.67	
*P*-value** (effect of treatment)		0.53	0.004	0.25	–	
Surrounding tissues	Week 0	80.22 ± 19.90	81.59 ± 13.57	77.27 ± 12.50	67.72 ± 16.67	<0.001
	Week 1	77.72 ± 22.92	82.27 ± 13.06	78.18 ± 11.90	70.90 ± 15.08	
	Week 2	85.68 ± 18.37	86.59 ± 12.2	79.77 ± 11.49	75.14 ± 14.14	
	Week 3	87.27 ± 13.69	87.14 ± 11.24	88.40 ± 16.15	80.00 ± 13.36	
	Week 4	89.28 ± 10.98	89.52 ± 11.05	82.04 ± 10.87	80.68 ± 20.83	
	Week 5	91.42 ± 8.36	91.31 ± 9.83	81.90 ± 11.45	87.72 ± 8.96	
	Week 6	92.42 ± 6.85	92.50 ± 8.14	81.90 ± 11.45	91.75 ± 6.54	
	Week 7	95.93 ± 5.23	96.33 ± 6.22	82.38 ± 11.57	96.11 ± 4.04	
	Week 8	98.92 ± 2.89	94.54 ± 5.68	82.61 ± 11.46	98.52 ± 2.93	
*P*-value** (effect of treatment)		0.057	0.54	0.038	–	
Drainages	Week 0	85.45 ± 3.70	90.15 ± 15.73	82.00 ± 24.76	80.68 ± 20.48	<0.001
	Week 1	87.50 ± 1.82	92.27 ± 10.54	85.68 ± 18.66	82.50 ± 19	
	Week 2	90.22 ± 10.05	95.22 ± 8.51	87.72 ± 16.67	84.54 ± 17.10	
	Week 3	94.54 ± 7.54	96.19 ± 7.40	88.40 ± 16.85	88.18 ± 14.92	
	Week 4	95.71 ± 6.38	69.66 ± 7.30	89.54 ± 17.03	92.72 ± 10.31	
	Week 5	97.36 ± 5.36	97.36 ± 6.31	89.52 ± 16.80	95.68 ± 8.20	
	Week 6	97.64 ± 4.37	97.18 ± 6.31	89.76 ± 16.84	89.00 ± 4.10	
	Week 7	99.06 ± 2.71	98.00 ± 5.60	89.76 ± 16.84	95.44 ± 15.79	
	Week 8	100.00 ± 0.0	98.33 ± 5.77	90.47 ± 15.56	95.52 ± 16.03	
*P*-value** (effect of treatment)		0.17	0.048	0.61	–	<0.001
Total ulcer status		304.31 ± 40.68	316.59 ± 33.18	300.63 ± 31.81	284.54 ± 45.77	
		309.09 ± 40.31	320.68 ± 28.96	307.27 ± 27.84	292.27 ± 39.99	
		328.86 ± 35.52	339.77 ± 26.4	313.18 ± 27.53	305.45 ± 36.93	
		345.45 ± 32.25	344.52 ± 24.43	318.86 ± 29.51	324.09 ± 33.08	
		353.57 ± 29.11	352.50 ± 28.35	324.31 ± 3.74	337.72 ± 35.86	
		363.94 ± 21.38	364.47 ± 26.18	323.80 ± 30.03	354.54 ± 23.54	
		370.58 ± 16.28	366.87 ± 24.21	324.52 ± 29.74	371.50 ± 16.86	
		382.05 ± 9.96	375.66 ± 23.66	327.85 ± 31.12	382.05 ± 9.69	
		392.50 ± 8.71	376.82 ± 25.12	367.82 ± 35.66	390.31 ± 13.84	
*P*-value** (effect of treatment)		0.081	0.024	0.048	–	

^*^Reference class–GEE analysis.

^**^Compared to the reference class.

Table 3 shows the mean scores of the tissue surrounding the wound of patients in the 4 groups over a period of 0–8 weeks. According to the results of GEE analysis, the effect of time was significant (*P*-value < 0.001), with the mean scores in the three intervention groups having an upward trend, but this trend was initially upward in the control group until it reached its peak at the seventh week and then it became downward in Week 8. Also, between-group comparison revealed that Group 15% was significantly different from the control group (*P*-value = 0.038).

Table 3 shows the mean scores of wound discharge of patients in the 4 groups over a period of 0-8 weeks. According to the results of GEE analysis, the effect of time was significant (*P*-value < 0.001), with the mean scores in all four groups having an upward trend. Also, between-group comparison demonstrated that Group 5% was significantly different from the control group (*P*-value = 0.048).

Table 3 shows the mean scores of the overall wound status of patients in the 4 groups over a period of 0–8 weeks. According to the results of GEE analysis, the effect of time was significant (*P*-value < 0.001), and the mean scores in the four groups had an upward trend. Also, between-group comparison showed that Group 15% and Group 5% were significantly different from the control group (*P*-value = 0.048, *P*-value = 0.024, respectively).

## Discussion

The aim of this study was to investigate the effect of a gel made with AF formulation on the healing of diabetic foot ulcers. Based on the findings of the present study, the most frequent wound site in the patients of the 4 groups was on the toes, while the least frequent one was on the whole foot. In studies by Nasiri et al. (6), most of foot ulcers were reported to be on the toes, which is consistent with the findings of the present study. However, the results of Aziza et al. (21) show that foot ulcers of 96% and 96% of their patients in the intervention and control groups were on the plantar surface, respectively, which is different from the results of the present study. Also in the present study, most patients had grade 2 ulcers according to Wagner classification.

One of the findings of the present study was to compare the overall healing of diabetic foot ulcers in the three treatment groups using AF gel at 5%, 10% and 15% doses (Group 5%, Group 10%, and Group 15%) and the placebo group before and after 8 weeks of intervention. The results showed that the AF gel, at different concentrations, was somewhat effective in the healing of diabetic foot ulcer, with gels at concentrations of 5% and 15% having the greatest effect on the healing process of diabetic foot ulcer compared to the 10% concentration.

On the other hand, the placebo which consisted of pharmacologically inert constituents had no effect on healing rate of wounds. In fact, like any other pharmaco-clinical study, the placebo was used to determine the real effect of test groups (AF groups). Such formula for chosen for the placebo so that it will have a non-irritating effect on tissue and be a good vehicle for AF in terms of compatibility and stability of AF gel formulation. The results of this study clearly depicted that the placebo had no real healing effect, and the wound healing effect is mainly due to the healing properties of the AF gels. Constituent of carrier gel including propylene glycol is being used as an excipient for a long time as it has subject to a report by European Medicine Agency entitled: Propylene glycol used as an excipient (22, 23). Regarding the HPMC Many techniques are available for studying the sol-gel transitions in HPMC hydrogels (24, 25). In fact this ingredient help to form a gel in association with propylene glycol. Both of them had no considerable effect on wound healing.

In line with the present finding, Alamouti et al. evaluated the safety of amniotic membrane extract in the healing of diabetic foot ulcers. Their results showed that the rate of wound healing during 4 weeks of treatment with amniotic membrane extract in the group of wounds with a size of 500 mm^2^ ≥ was 98.9 ± 2.40%, and in wounds with a size of 500 mm^2^ ≤, it was 92.1 ± 7.23%, which indicates the effect of amniotic membrane extract on the healing of diabetic foot ulcers (17). Also, the effect of human amniotic membrane on reducing the size of foot ulcers in diabetic patients has been repeatedly reported in clinical trial studies (17, 26, 27). In the study of Zelen et al., for example, the mean duration for complete wound healing with Epifix was 23.6 days (27). Epifix is a proprietary product for wound healing, containing dehydrated amnion and chorion membrane. It is used in wound clinics and produced by MiMedix Co, USA.

With respect to diabetic foot ulcer discharge in the four studied groups, the results indicate that amniotic fluid gel at different concentrations was somewhat effective in the reduction of diabetic foot ulcer discharge, but gel with 5% concentration had the greatest effect in treating diabetic foot ulcer discharge compared to 10% and 15% concentrations. Consistent with the present finding, Hakim et al. (20) found a significant difference between the two groups in terms of wound discharge in 4 weeks of treatment. However, in Nasiri et al., no significant difference was reported between the experimental and control groups in terms of wound discharge after 4 weeks of intervention (6). One of the reasons for the difference between our results and those of Nasiri et al. is the different type of intervention.

As far as the tissue surrounding diabetic foot ulcer is concerned, the AF gel at 5, 10 and 15% doses was effective before and after 8 weeks of intervention compare with the placebo group. However, the gel at the concentration of 15% had the greatest effect on the discharge reduction of diabetic foot ulcers. In Hakim et al. (20) and Nasiri et al. (6), a significant difference was observed between the two groups in terms of tissues surrounding the wound in weeks 2, 3, and 4 of treatment, which is in line with the results of the present study.

Apropos of the color of diabetic foot ulcer in four studied groups, our results showed that amniotic fluid gel at different concentrations affected diabetic foot ulcer color, but gel with 5% concentration had the greatest effect on the wound color in diabetic foot ulcers. In Hakim et al. (20) and Nasiri et al. (6), there was a significant difference between the two groups in terms of wound color after 4 weeks of treatment, which is completely consistent with the results of the present study.

Finally, the results of the present study revealed that amniotic fluid gel at different concentrations had a significant effect on the grade of diabetic foot ulcer, but the gel at 5% concentration had the greatest effect on the process of diabetic foot ulcer. In Nasiri et al., the wound grade score at the end of weeks two, three and four was significantly higher than that in the previous week, which indicates the upward trend of wound healing (6). In Hakim et al. (20), there was a significant difference between the two groups in terms of wound grade after 4 weeks of treatment.

The main limitation of the present study was that the process of wound healing is different in different people due to their genetic profile, and this might have affected the results but was beyond the control of the researchers.

## Conclusion

According to the results of the present study, amniotic fluid gel at different concentrations has a significant effect on the grade of diabetic foot ulcer. Given these patients' special condition and their urgent need for various trainings in relation to their disease and the associated interventions, it is necessary that nurses and even all members of the health team attend patient training programs aimed at orienting them on how to care for and prevent diabetic foot ulcers. Also, the use of effective and available materials such as the amniotic gel used in this study should be promoted in order to treat foot ulcers of diabetic patients and thus reduce the rate of amputation and the physical, mental, social and economic complications associated with their condition.

## Guarantor's statement

SM is the guarantor of this work and, as such, had full access to all the data in the study and takes responsibility for the integrity of the data and the accuracy of the data analysis.

## Data availability statement

The original contributions presented in the study are included in the article/supplementary material, further inquiries can be directed to the corresponding author.

## Ethics statement

The studies involving human participants were reviewed and approved by Ahvaz Jundishapur University of Medical Sciences. The patients/participants provided their written informed consent to participate in this study.

## Author contributions

FN, SM, DB, AH, and LY researched data. FN, SM, DB, AH, LY, AS, MM, and NS wrote the manuscript and researched data. FN and SM contributed to discussion and reviewed and edited the manuscript. All authors contributed to the article and approved the submitted version.
